# A nomogram to predict the prognosis of patients with unresected rectal adenocarcinoma undergoing chemoradiotherapy: a population-based study

**DOI:** 10.7150/jca.61642

**Published:** 2021-06-11

**Authors:** Lin-Lin Liu, Jun-Die Sun, Zuo-Lin Xiang

**Affiliations:** Department of Radiation Oncology, Shanghai East Hospital, School of Medicine, Tongji University, Shanghai, 200120, China.

## Abstract

**Background:** Chemotherapy combined with radiotherapy is the main treatment strategy for unresectable rectal cancer. However, the prognostic factors of patients with unresectable rectal cancer treated with radiotherapy and chemotherapy have not been systematically studied. Therefore, this study investigated the prognostic factors and prognosis based on surveillance, epidemiology and final results of the SEER medical insurance database.

**Methods:** Primary rectum patients were selected from the SEER database. The independent prognostic factors associated with overall survival (OS), cancer-specific survival (CSS) and noncancer-related death were evaluated using the Kaplan-Meier method and log-rank test, a competing risk model, and the Cox proportional hazards regression model. Two nomograms were established for predicting the 1- and 3-year OS and CSS of patients.

**Results:** A total of 3,998 rectal adenocarcinoma cancer patients who received chemoradiotherapy but had not undergone surgery were included in this study and divided into training (n = 3559) and validation cohorts (n = 439). Patients in the training cohort had a 1-year OS rate of 65.7±0.8%, a 3-year OS rate of 26.6±0.8%, a 5-year OS rate of 1.6±0.8%, and a median survival rate of 20.0 months (range, 19.22-20.8 months). The following factors were significant prognostic factors of OS: age (p< 0.001); tumour grade (p< 0.001); T stage (p< 0.001); M stage (p< 0.001); bone metastasis (p<0.001); brain metastases (p<0.001); liver metastases (p<0.001); lung metastases (p<0.001); marital status (p<0.001) and insurance status (p=0.005). Age (p< 0.001), tumour grade (p< 0.001), T stage (p< 0.001), M stage (p< 0.001), bone metastasis (p<0.001), brain metastases (p<0.001), liver metastases (p<0.001), lung metastases (p<0.001) and race (p=0.034) were independent prognostic factors of CSS. Age (p< 0.001), T stage (p< 0.001), N stage (p=0.007), M stage (p<0.001), liver metastases (p<0.001), lung metastases (p<0.001), marital status (p<0.001) and insurance status (p=0.019) were independently associated with noncancer-related death.

**Conclusion:** Age, tumour grade, T and M stage, bone, brain, liver and lung metastases, marital status and insurance status are independent risk factors for the OS of rectal adenocarcinoma patients who have undergone chemoradiotherapy but have not undergone surgery. Age, tumour grade, T stage, M stage, bone, brain, liver, lung metastases and race were independent prognostic factors of CSS. Age, T, N and M stage, liver and lung metastases, marital status and insurance status, were independently associated with noncancer-related death. Interestingly, the earlier the T stage was, the higher the rate of noncancer-related death. Two nomograms were developed to predict OS and CSS, and the C-indexes were 0.6776 and 0.6744, respectively. Rectal cancer screening is strongly recommended for patients under the age of 50.

## Introduction

Rectal cancer is one of the most common malignant tumours of the digestive system in clinical practice, and its morbidity and mortality rates are among the top 5 in the world, showing increasing trends year by year 1. In 2019, an estimated 44,180 new cases of rectal cancer were diagnosed in the United States. The incidence of rectal cancer in the European Union is between 15 and 25 cases per 100,000 people, and approximately 33% of these cases result in death each year 1-3. In 1979, the World Health Organization introduced the classification of colorectal cancers according to their histology, which were defined as classical adenocarcinomas (ACs), which account for the large majority of cases; mucinous adenocarcinomas (MACs); signet-ring cell carcinomas (SCs); and other less frequent forms 4.

Although rectal cancer is more common among elderly individuals, a large number of studies have shown a significant increase in the incidence of rectal cancer in young people. The diagnosis of patients under the age of 50 (from 1974 to 2010) has increased significantly, and it is predicted that by 2030, the incidence of rectal cancer in patients aged 20-34 will increase by 124.2% 5.

Compared to cancer in the more proximal large intestines, mid- and lower rectal cancer was associated with higher rates of local recurrence and reduced disease-free survival 6. Overall, due to differing embryologic aetiologies, lymphovascular drainage basins, and molecular mutational burdens, even between the sigmoid colon and rectum 7, 8, rectal cancer has a higher frequency of metastases and local recurrence than colon cancer, which means a worse prognosis 9.

It is generally believed that to reduce the local recurrence rate and improve the long-term survival rate, in conjunction with radiation, curative intent rectal surgery with total mesorectal excision (TME) is standard 10, 11. Among them, for stage II (T3-4, lymph node negative) and stage III (lymph node positive) patients, NCCN recommends neoadjuvant CRT, followed by surgical resection and then adjuvant chemotherapy. The total perioperative course of treatment should not exceed 6 months 12. However, there are many surgical complications, including wound infection, intra-abdominal abscess, sepsis (incidence rate of 12%), anastomotic leakage (incidence rate of 11%), and apostoperative mortality rate of 2%; additionally, the patients also have to bear the risk of organ dysfunction and incontinence 13-17.

Therefore, although it is not the standard treatment, an increasing number of people prefer the nonsurgical treatment strategy for rectal cancer, that is, to choose radiotherapy and chemotherapy first 18.

Radiation therapy is the targeted administration of X-rays. The accumulation of radiation-mediated DNA breaks and ROS damage ultimately induces cell death 19. Chemotherapy can be used for systemic treatment, and radiotherapy combined with conventional chemotherapy, such as cisplatin and 5-FU, can improve the radiation response of rectal cancer 20-22.

This can be done for three purposes: for preoperative therapy, accidental watch-and-wait in cases where the tumour has a complete clinical response and intentional watch-and-wait 23-25. Additionally, because tumour regression is a time-dependent phenomenon, a series of retrospective data show that a longer interval response is beneficial to improve the possibility of achieving complete pathology 26, 27. The tumour outcome of patients with a complete clinical response to nonsurgical treatment was similar to that of patients with a complete pathological response to radical surgery 28. The work of Martens et al. confirmed the application of the watch-and-wait approach in the treatment of rectal cancer, with a 3-year overall survival rate of 97% and a distant metastasis-free survival rate of 97%. Other published analyses further confirm these observations and provide more reliable data to support these findings 29-34. A case report of stage IV rectal cancer also mentioned that only radiotherapy combined with chemotherapy was used to meet the CCR standard of endoscopic examination 35. Most of the previous studies have focused on the survival analysis of rectal cancer patients treated with neoadjuvant therapy; however, there are few reports on the prognosis and factors affecting the survival of patients with rectal adenocarcinoma who choose the combination of radiotherapy and chemotherapy. To fully understand the prognosis of patients with rectal adenocarcinoma cancer undergoing radiotherapy combined with chemotherapy and the independent factors that affect the prognosis, we analysed the medical records from the surveillance, epidemiology and final outcome database (http://seer.cancer.gov/) and developed two nomograms to directly predict the prognosis of these patients.

## Methods

### Study population

We used SEER * State software (version 8.3.5, National Cancer Institute, Bethesda, Maryland) to retrospectively extract data from the SEER database from 2004 to 2016, the SEER database, which is one of the largest population-based cancer registry programs in the United States, covering 28% of the U.S. population at 18 cancer registry centres. Since the data collected from the SEER database were anonymous prior to publication, patient informed consent was not required in our study.

**Figure 1:**
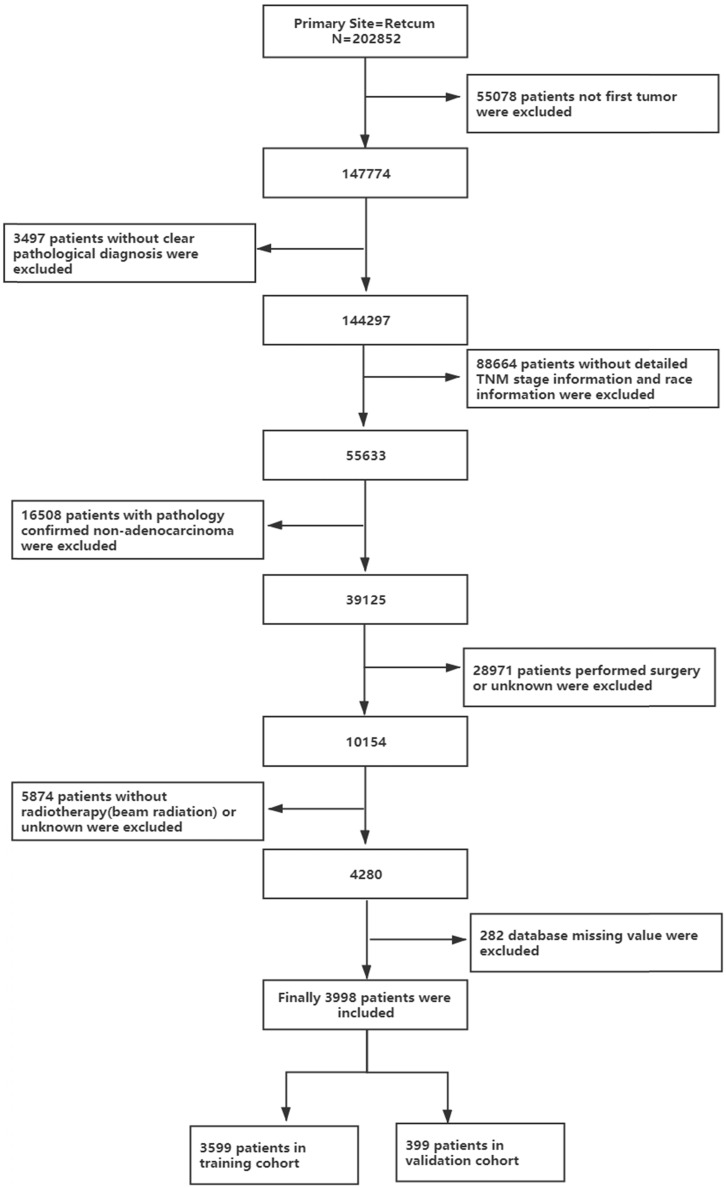
The flow diagram of the selection process for the study.

All authors have obtained permission from SEER to access the original data. This study included all patients with rectal adenocarcinoma who received chemotherapy and radiotherapy ("rectal" was ICD-O-3/WHO 2008, "malignant" was ICD-O-3, and behaviour was ICD-O-3). The inclusion criteria were as follows: (1) diagnosed as the first and only rectal adenocarcinoma; (2) complete demographic characteristics and survival information; (3) tumour staging according to AJCC 6th or 7th stage; (4) received combined radiotherapy and chemotherapy; and (5) nonsurgical treatment. The process is shown in Figure 1. Patient diagnosed before 2004 were excluded because they did not receive the 6th edition of the AJCC staging system.

### Variable selection

We extracted various determinants from the SEER database. For each record, we counted the demographics, tumour variables and follow-up data. The sociodemographic data involve race, sex, age, marital status, and insurance status. The tumour parameters include the degree of tumour differentiation, tumour size, AJCC stage, T stage, N stage, M stage, clinical stage, and the extent of distant metastatic sites involving bone, brain, liver and lung at primary diagnosis of rectal adenocarcinoma. The follow-up data contain cause of death, status, and survival time from the SEER database. Among them, we classified patients by age (18-49, 50-59, 60-100) and tumour size (<5 mm and ≥5 mm) to facilitate the calculation of the cumulative incidence function (CIF). Race was grouped as white, black, and others, whereas marital status was categorized into married, single and unknown. Insurance status was classified as insured uninsured and unknown. The 7th edition of the AJCC staging system was applied to the patients in this study. Histology of rectal cancer was adenocarcinoma.

Overall survival (OS) was the primary endpoint, and cancer-specific death was the secondary endpoint, which was defined as the period from diagnosis to death caused by rectal cancer or censoring. In addition, the study includes two groups. Ninety percent of patients were randomly divided into the training cohort, and ten percent of patients were classified into the validation cohort.

### Statistical analysis

Descriptive statistics were used to examine the following baseline characteristics of cases. The sociodemographic data (race, sex, insurance status and marital status) and the tumour parameters (degree of tumour differentiation, tumour size, AJCC stage, T stage, N stage, M stage, clinical stage and extent of distant metastatic site involving bone, brain, liver and lung at primary diagnosis of rectal cancer).

For the survival analysis and prognosis evaluation, the cases were divided into 2 groups by random sampling. In the training cohort, the Kaplan-Meier method was first used to draw the survival curve of each clinicopathological factor, and the log-rank test was used for comparison.

Second, to validate that significant variables were assessed through competitive risk analysis, we treated other causes of death as the competing event of cancer-specific death in early-onset rectal adenocarcinoma. The CIF was calculated to estimate the probability of cancer-specific death at different time points. We plotted the CIF curves for each variable and performed Gray's test to recognize the differences in cancer-specific mortality among different subgroups. Then, a multivariate analysis was conducted through a Cox regression model.

Finally, we built 2 nomograms based on the results of multivariate analysis. Overall survival (OS) and cancer-specific survival (CSS) rates were predicted with the help of nomograms. The performance of the nomogram was internally measured by the concordance index (C-index), which was assessed by comparing the nomogram-predicted probability with the observed probability.

## Results

### Demographic and clinical characteristics

As shown in Figure 1, this study finally selected 202,852 patients with rectal cancer diagnosed between 2004 and 2016, of whom 3,998 pathological types of adenocarcinoma received chemoradiotherapy instead of surgery, accounting for 1.97% of the total. As shown in Table 1, the mean follow-up time for the entire cohort was 28.518 months. The ratio of males to females was 1:1.7182, which reflects the higher number of males entering the program, and most of the patients were white (77.2%). The median age of the patients was 63.4 years. Regarding tumour grading, 61.7% of rectal adenocarcinomas were moderately differentiated (G2), 10.9% were poorly differentiated (G3) and 6.6% were well differentiated (G1). In terms of tumour stage, 8.7% of tumours were stage I, 24.5% of tumours were stage II, 25.1% of tumours were stage III and most patients were grade IV (41.7%). More than half of the patients (58.3%) were in stage M0. In terms of tumour size, 60.2% of tumours were less than 5 cm in diameter, and 27.9% of tumours were greater than or equal to 5 cm in diameter. Most patients were insured (65.8%), and 46.5% of them were married.

### Survival analysis

As shown in Figure 1, of the 39,125 patients with rectal adenocarcinoma, 3,998 were treated with a combination of radiotherapy and chemotherapy instead of surgery, and 3,599 patients were included in the training cohort. From Table 1, we know, until the end of follow-up, the median survival time of the training cohort was 20 months (range, 19.179-20.821 months), with a 1-year overall survival of 65.7±0.8%, a 3-year overall survival of 26.6±0.8% and a 5-year overall survival of 15.4±0.9%.

**Figure 2:**
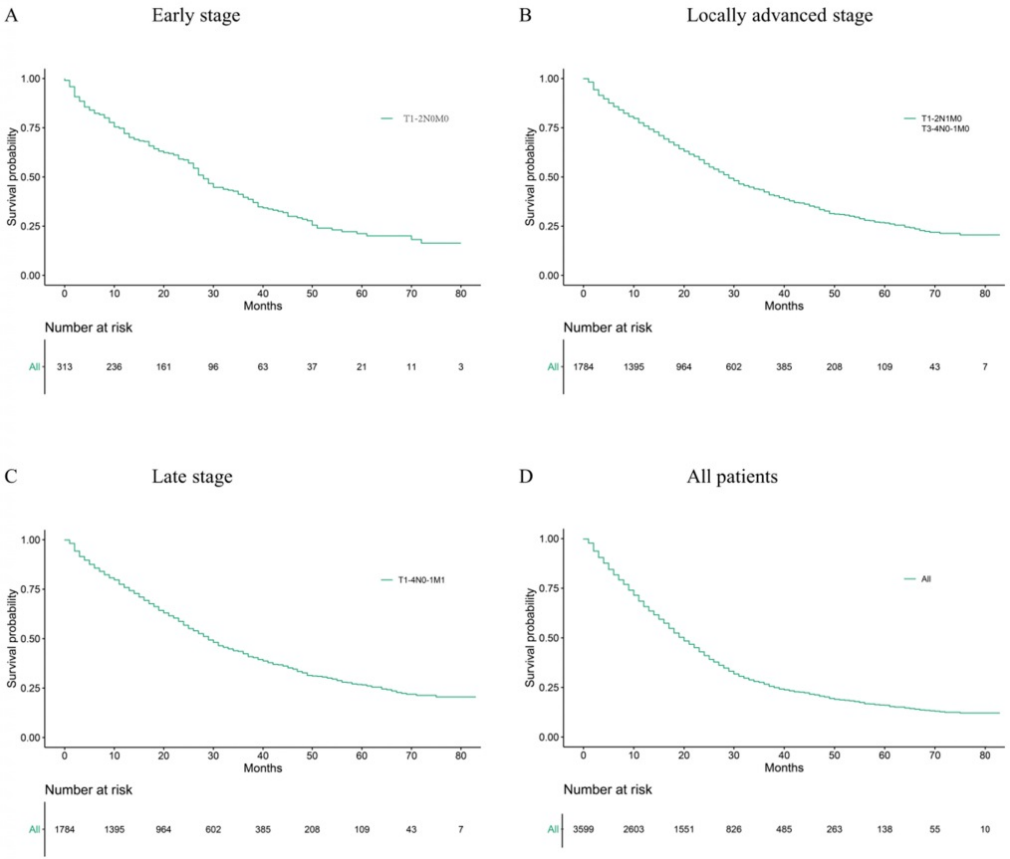
Kaplan Meier analysis for OS in patients with different tumour stages (A) Early stage, (B) Locally advanced stage, (C) Late stage, (D) All patients.

Median age at diagnosis of cancer is 63.44 years old. The rectal adenocarcinoma patients in the training group were divided into early stage, locally advanced stage and late stage, which included 313, 1784 and 1502 patients, respectively. Figure 2 and Table 2 show the 1-year, 3-year and median survival time and survival curves.

**Figure 3:**
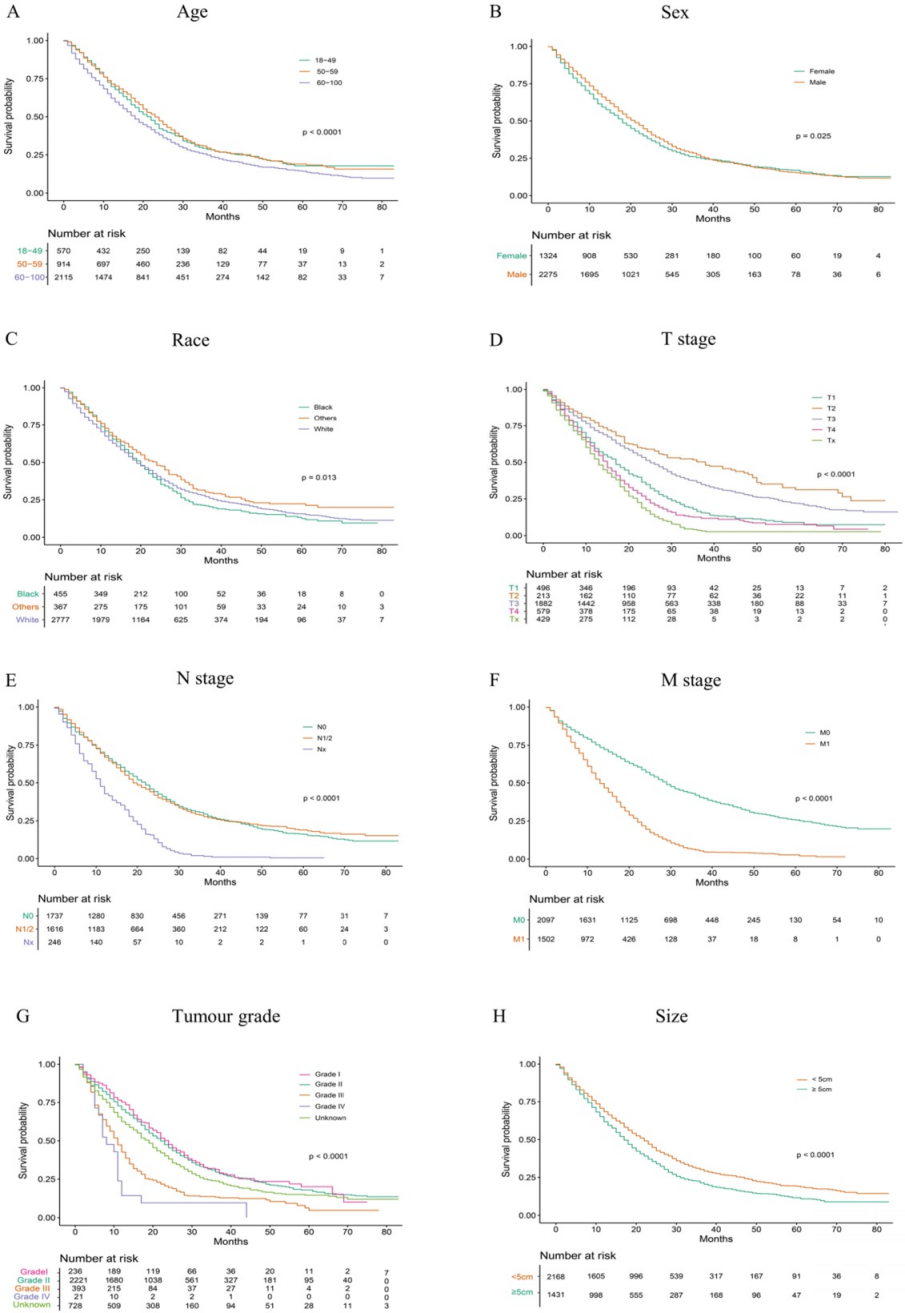

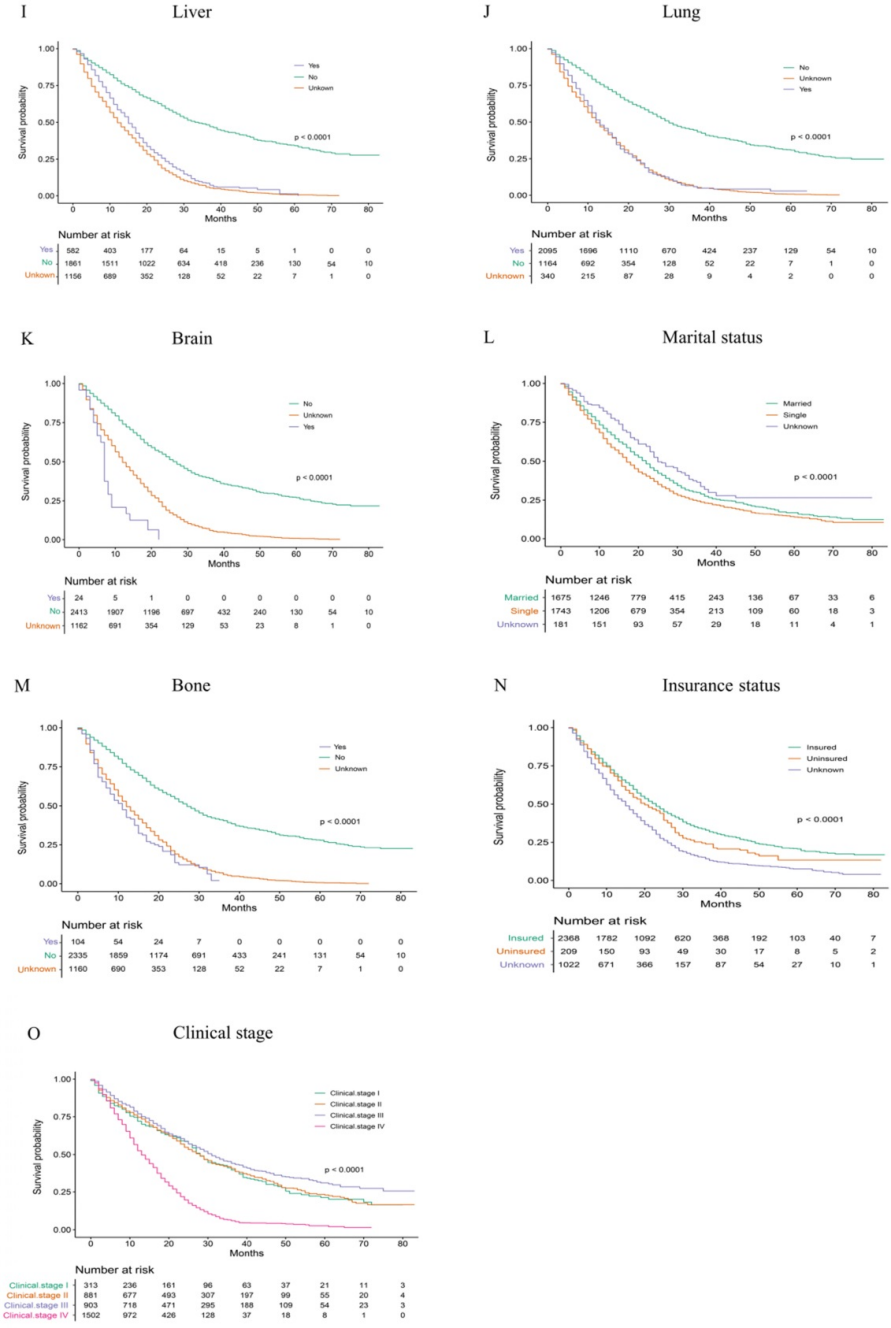
Overall Kaplan-Meier survival curves for patients in training cohort according to (A) Age, (B) Sex, (C) Race, (D) T stage, (E) N stage, (F) M stage, (G) Tumour grade, (H) Size, (I) Liver, (J) Lung, (K) Brain, (L)Marital status, (M) Bone, (N) Insurance status, (O) Clinical stage.

**Figure 4:**
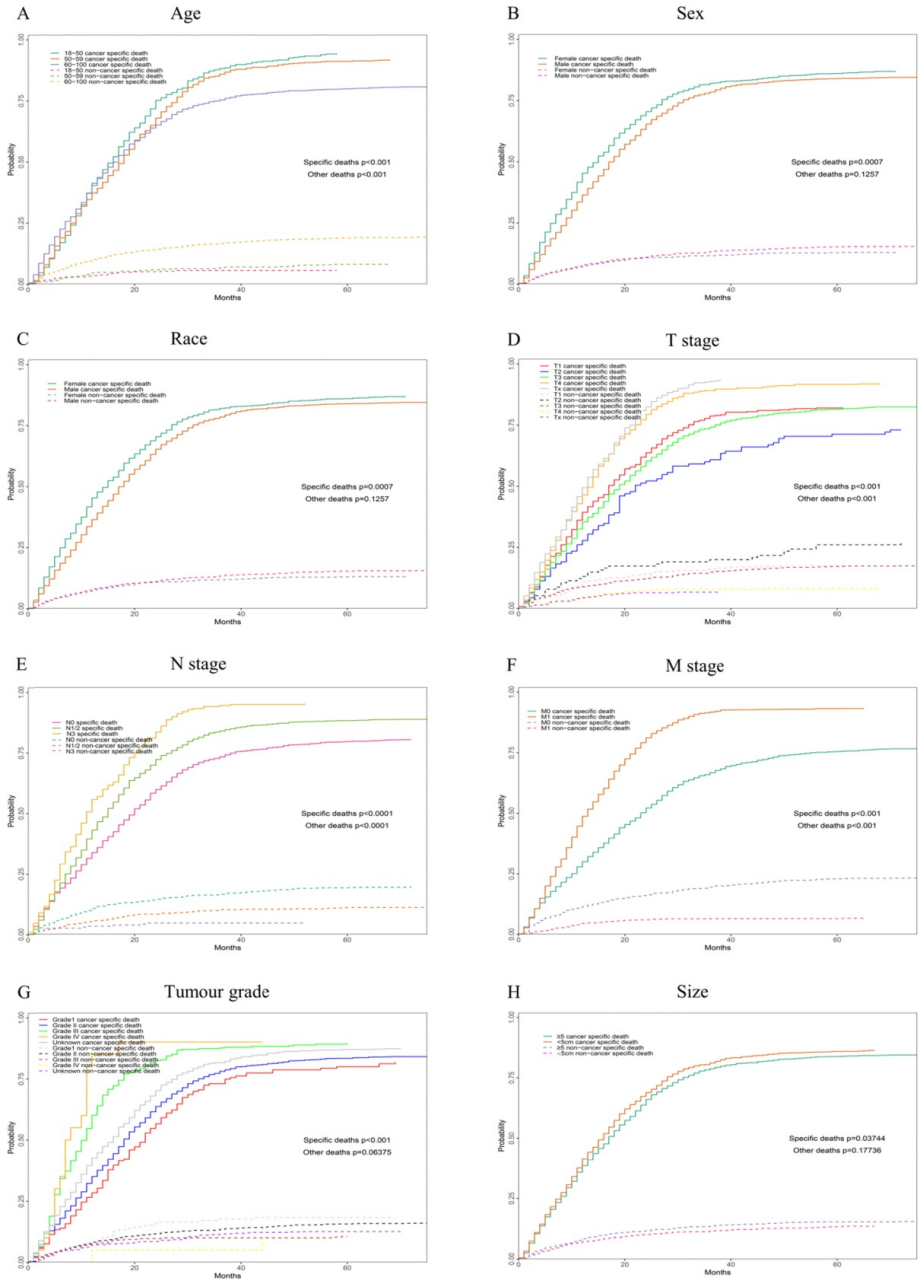

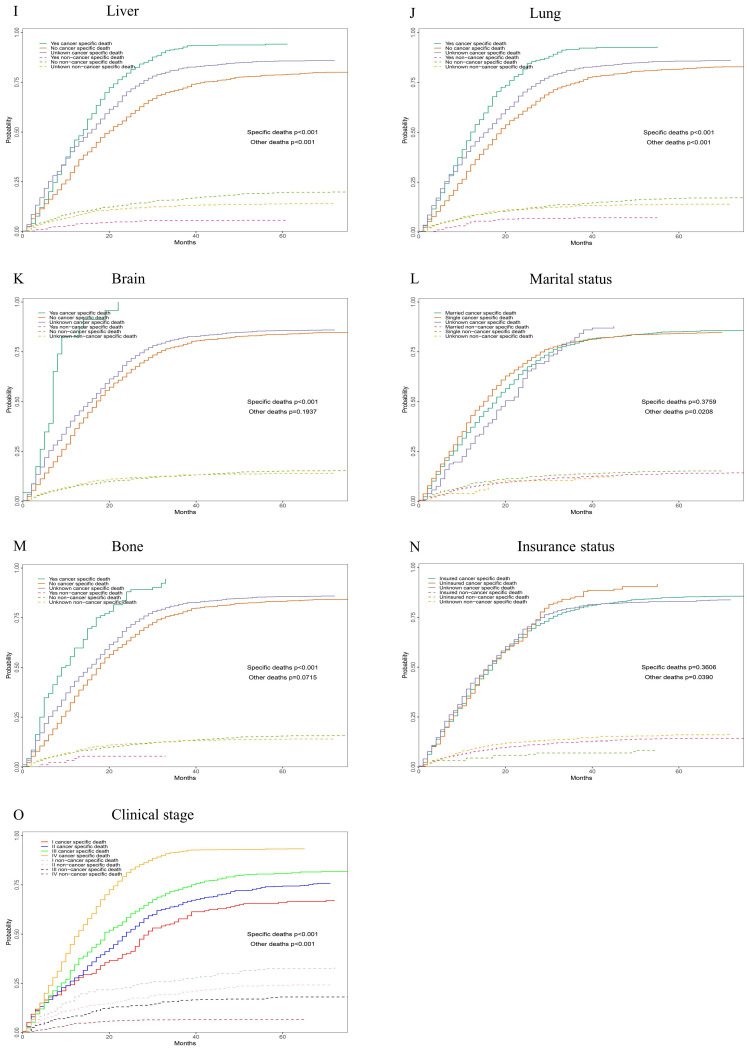
Competing risk analyses for patients in training cohort according to (A) Age, (B) Sex, (C) Race, (D) T stage, (E) N stage, (F) M stage, (G) Tumour grade, (H) Size, (I) Liver, (J) Lung, (K) Brain, (L)Marital status, (M) Bone, (N) Insurance status, (O) Clinical stage

According to our univariate analysis model, as shown in Figure 3 and Table 3, age (*p*<0.001), race (*p*=0.027), pathologic grade (*p*<0.001), T stage (*p*<0.001), N stage (*p*<0.0011), M stage (*p*<0.001), bone metastasis (*p*<0.001), brain metastases (*p*<0.001), liver metastases (*p*<0.001), lung metastases (*p*<0.001), marital status (*p*<0.001), insurance status (*p*<0.001), tumour size (*p*<0.001), sex (*p*<0.001) and TNM stage (*p*<0.001) were significant prognostic factors of OS. Furthermore, the analysis of the cancer-specific survival curve based on the competitive risk model is summarized in Figure 4 and Table 4. The results were similar to those of the univariate analysis but excluded the variables race (*p*=0.716), marital status (*p*=0.3759) and insurance status (*p*=0.3606). Additionally, age (*p*<0.001), TNM stage (*p*<0.001), T stage (*p*<0.001), N stage (*p*<0.001), M stage (*p*<0.001), liver metastases (*p*<0.001), lung metastases (*p*<0.001), insurance status (*p*=0.039), and marital status (*p*=0.021) were considered to be related to noncancer-related death, as shown in Table 4.

Finally, the following variables included in the regression equation of the predicted OS, CSS and noncancer-related death were determined by Cox proportional hazard regression. In this step, internal variables, including N stage (*p*=0.469), size (*p*=0.495), sex (*p*=0.621) and race (*p*=0.082), were considered not significantly related to OS. The regression equation for predicting CSS included pathologic grade (*p*<0.001), T stage (*p*<0.001), M stage (*p*<0.001), bone metastasis (*p*<0.001), brain metastases (*p*<0.001), liver metastases (*p*<0.001), lung metastases (*p*<0.001), age (*p*<0.001) and race (*p=*0.034). In addition, the regression equation for predicting noncancer death included T stage (*p*<0.001), N stage (*p*=0.007), M stage (*p*<0.001), liver metastases (*p*<0.001), lung metastases (*p*<0.001), marital status (*p*<0.001) and insurance status (*p*=0.019).

### Construction and validation of the nomogram

**Figure 5:**
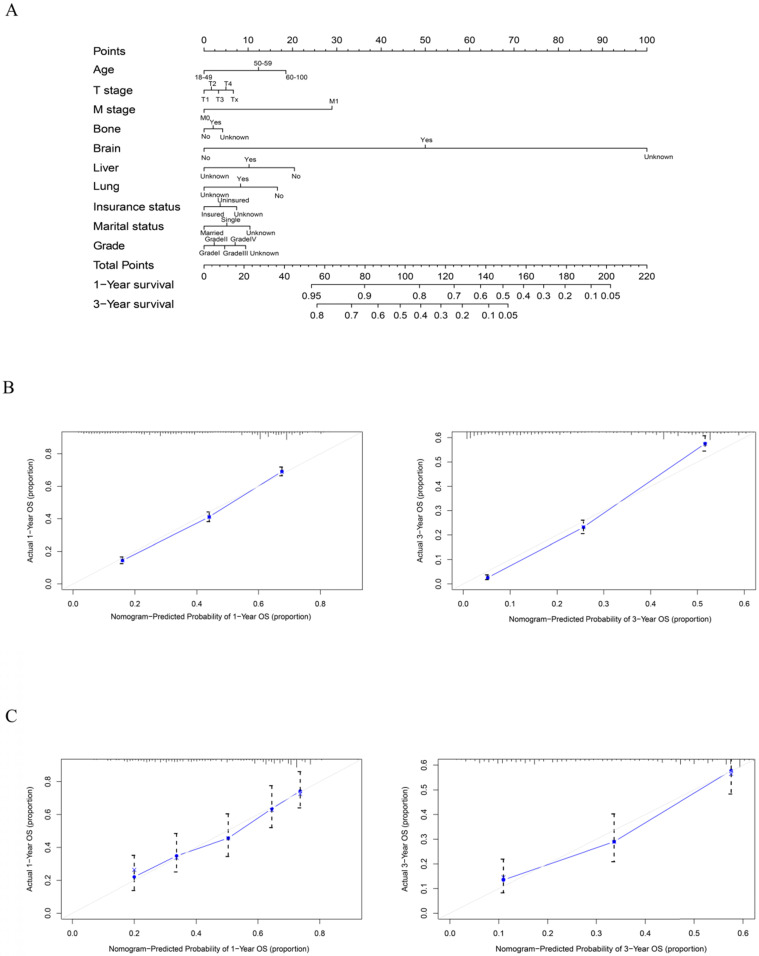
A nomogram for prediction of 1- and 3-year OS rates of rectal adenocarcinoma patients who received chemoradiotherapy but had not undergone surgery (A); Calibration curve of the nomogram predicting 1- and 3-year OS rates of rectal adenocarcinoma patients who received chemoradiotherapy but had not undergone surgery in training cohort (B); Calibration curve of the nomogram predicting 1- and 3-year OS rates of rectal adenocarcinoma patients who received chemoradiotherapy but had not undergone surgery in the validation cohort (C).

**Figure 6:**
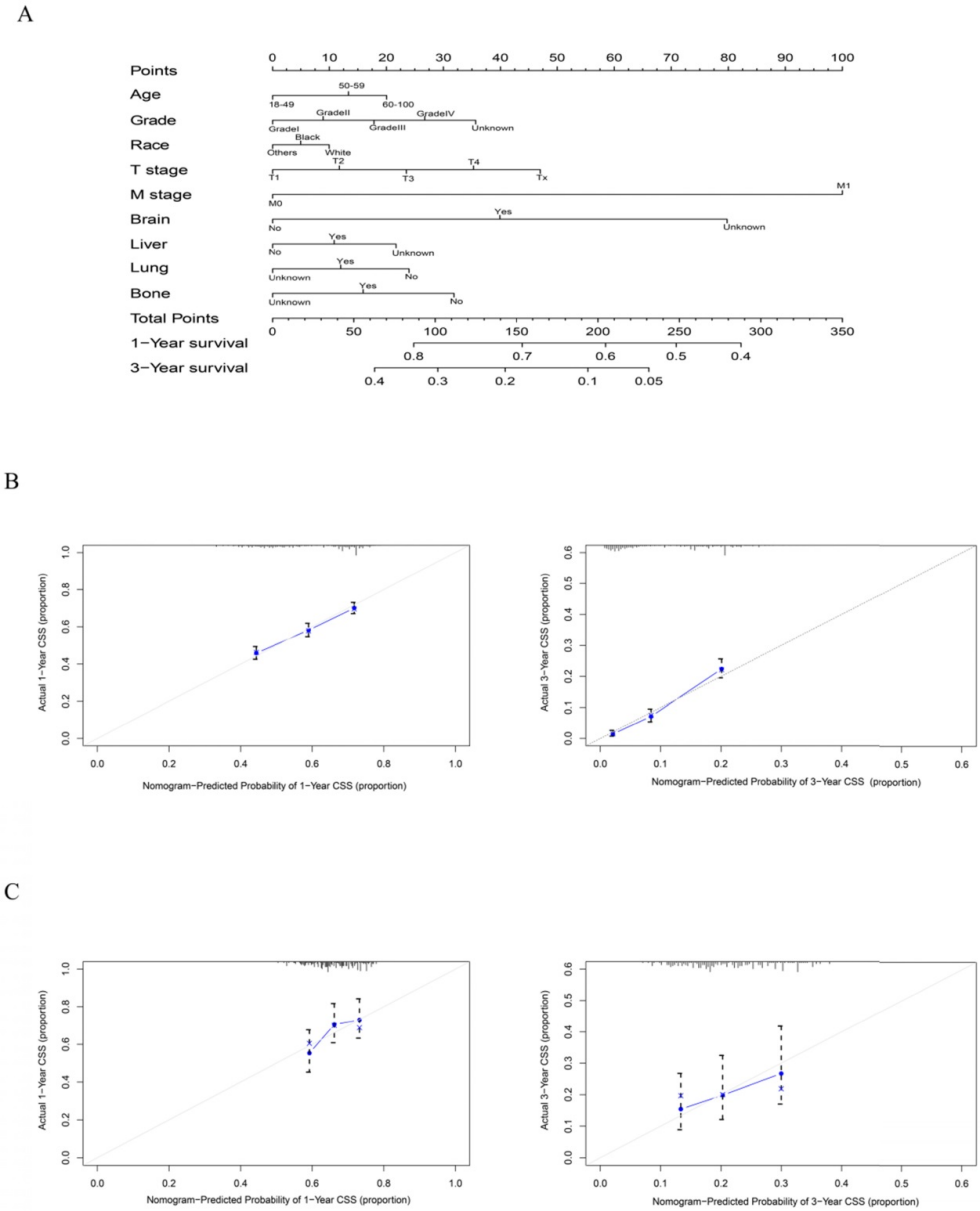
A nomogram for prediction of 1- and 3-year CSS rates of rectal adenocarcinoma patients who received chemoradiotherapy but had not undergone surgery (A); Calibration curve of the nomogram predicting 1- and 3-year CSS rates of rectal adenocarcinoma patients who received chemoradiotherapy but had not undergone surgery in training cohort (B); Calibration curve of the nomogram predicting 1- and 3-year CSS rates of rectal adenocarcinoma patients who received chemoradiotherapy but had not undergone surgery in the validation cohort (C).

As shown in Figure 5, the final nomogram was formulated with age, grade, T stage, M stage, bone, brain, liver and lung metastasis, marital status and insurance status and showed the 1- and 3-year OS by weighting the score of each variable. As shown in Figure 6, another nomogram that predicts the 1- and 3-year CSS was depicted as follows. The final nomogram was formulated with age, grade, T stage, M stage, bone, brain, liver and lung metastasis, and race, which was shown by weighting the score of each variable. The nomograms were both internally verified through identification and calibration methods, and the calculated C-indexes were 0.6776 and 0.6744, respectively, indicating that the models are in good agreement with the actual observation results. The verification queue data were used for the external verification of the nomogram, and the predicted survival rates of the nomogram established by the verification queue were comparable to the actual survival rate of the patient. Therefore, two nomograms are reliable.

## Discussion

The study included 202,852 patients with rectal adenocarcinoma diagnosed from 2004 to 2016 and analysed their survival. Through statistical tests, we evaluated independent prognostic factors affecting OS, CSS and noncancer-related death in 3,599 patients with adenocarcinoma who received chemoradiotherapy instead of surgery. Then, two nomograms were constructed to quantitatively evaluate the 1- and 3-year survival rates using the above factors. A total of 399 patients in the verification set were used for external verification, and internal verification was also performed to evaluate the accuracy and validity of the nomogram. Among all rectal adenocarcinoma patients, liver metastases accounted for 51.7%, lung metastases accounted for 58.2%, bone metastases accounted for 64.9%, and brain metastases accounted for 67.0%.

It is well known that TNM staging is an independent prognostic factor for rectal survival, and has a stronger correlation with rectal cancer mortality 36. However, the clinical guidelines of the AJCC Cancer and Lymph Node Metastasis (TNM) staging system of the American Joint Commission on Cancer are based only on biological factors, excluding nonbiological factors (NBFs) 37. Based on the clinical management and prognostic accuracy of rectal cancer, we evaluated the clinical value of including NBF in the TNM staging system.

There is no doubt that age is a prognostic factor for most tumours. The same is true for patients with rectal cancer. However, in the last decade, the incidence of rectal cancer has increased exponentially in patients less than 50 years of age, even without a family history of colorectal cancer 38. To further clarify the causes of this phenomenon and identify potential prevention and early detection strategies. We stratified the age; that is, 3599 patients were divided into three groups according to their age, accounting for 15.8%, 25.4% and 58.8%, respectively. Univariate analysis showed that the 1-, 3-and 5-year median survival times of patients aged 50-59 were longer than those of patients aged 18-49 and 60-100 years. Multivariate analysis showed that age was an independent factor affecting the prognosis of OS, CSS and noncancer-related death. Why do young patients (18-49 years old) have worse survival than older patients (50-59 years old) when they receive the same treatment?

The reasons for consideration may be as followed:

1. Screening for ordinary people begins at the age of 50, so it may delay the early detection of rectal cancer in patients under the age of 50, thus delaying the disease 39.

2. The study found that 46, 47, 48 and younger patients were more likely to present with poorly differentiated and late-stage cancers 40.

At present, the focus of prevention and treatment of rectal cancer is to reduce the incidence of rectal cancer in the elderly and prolong survival, but the high incidence and poor prognosis of young patients with rectal cancer cannot be ignored. Although the young patients selected in this article cannot be represented as a group of young people with rectal cancer, they are all people who refuse surgery. However, it can still be observed that in this group of people, the prognosis of rectal cancer patients aged 18-49 is worse than that of 50-59 years old, so it is strongly recommended that rectal cancer screening be carried out for patients under 50 years old.

The findings of the present study showed that tumour size was not associated with OS, CSS or noncancer death, and trends towards increased OS and CSS were observed for patients with tumour size <5 cm compared with patients with a tumour size ≥5 cm.

Race is an independent factor of CSS, but it has no effect on OS and noncancer-related death. Multivariate Cox regression analysis revealed that marital status was an independent prognostic factor for OS, CSS and noncancer-related death. Although they are not independent prognostic factors of CSS, they are related to CSS.

The degree of tumour differentiation has an impact on OS and CSS, that is, the higher the grade of the tumour is, the worse the prognosis of the patient, which is not observed in noncancer-related death. In previous studies, it was found that patients with poorly differentiated rectal cancer had a poor prognosis, but in patients treated with chemoradiotherapy, we found that the better the differentiation was, the worse the prognosis was 41.

Unsurprisingly, T staging is an independent prognostic factor of OS, CSS and noncancer-related death. The literature on the prognosis of rectal cancer pointed out that in the case of specific treatment, the higher the T stage was, the worse the patient's prognosis was 42. This may be due to the increase in cT staging and the increased risk of local regeneration of rectal cancer. However, in rectal adenocarcinoma patients who choose chemoradiotherapy without surgery, the situation is not what we think.

In view of this phenomenon, we carried out observations and analysis. First, according to the statistical description, the median survival times at 1 year, 3 years and 5 years were as follows: stage T2> T3 >T1> T4. Second, the analysis of the multivariate Cox regression equation showed that T2 and T3 were protective factors against OS and CSS compared with T1. (0.583, 0.822 V.S. 1; 0.569, 0.817 V.S. 1). Third, according to the Finy-Gray test, the odds ratio (OR) of noncancer-related death and death at T1 was significantly higher than those at T2 and T3 (1 V.S. 0.548; 0.830), which means that T1 is a risk factor for noncancer-related death compared to T2 and T3.

The third test explains the reason for this phenomenon:

T1 patients are more likely to die due to some noncancer-related causes, thus affecting OS. For patients with rectal tumours staged as T1 or T2, surgery alone is the recommended standard of care. If no operation is performed, there must be reasons that prevent the patient from performing the operation, such as poor physical condition, severe hypertension or cardiovascular disease, resulting in surgical intolerance; that is, the patient's own poor health condition may affect survival. Therefore, considering the existence of noncancer-related death, to further clarify the actual survival situation related to cancer, we established a nomogram to predict CCS, as shown in Figure 6.

The analysis of the results of N staging showed that the survival condition of patients with N1 and N2 disease was worse than that of patients with N0 disease, regardless of OS, CSS or noncancer-related death. There is evidence that lymph node metastasis is the main predictor of the prognosis of patients with colorectal cancer 43, 44. It has been suggested that compared with the presence of 1-3 positive lymph nodes (N1 disease, 3-year disease-free survival rate of 83%) and N0 disease (3-year disease-free survival rate of 89%), 4 or more lymph node metastases (N2 disease) Of patients, the 3-year disease-free survival rate (75%) is lower 45. Therefore, whether it is to identify the presence or absence of lymph node metastasis or to accurately distinguish the number of metastatic lymph nodes is very valuable to the prognosis of patients with rectal cancer. Based on this, MRI is recommended to identify metastatic lymph nodes. It is the best way to identify lymph node diseases by predicting tumour invasion through signal heterogeneity and irregular borders 46. In addition, high-resolution MRI can improve diagnostic performance and is recommended as the best strategy for patient selection.

Multivariate Cox regression shows that M stage is an independent prognostic factor of the three survival styles. Typically, the most common sites of rectal cancer metastasis are regional lymph nodes, liver, lung, bone and brain 47. Haematogenous metastasis is very common in rectal cancer metastasis. However, in our study, 67% of people had liver metastases, 58.2% had lung metastases, 64.9% had bone metastases, and 67.0% had brain metastases, indicating that if bone metastases and brain metastases occur, patients are more inclined to not undergo surgery. The prognosis of rectal cancer patients with distant metastasis is poor. The 3-year survival rate of patients with bone or brain metastasis was almost zero, the 3-year survival rate of patients with lung metastasis was 0.059±0.015, and that of patients with liver metastasis was 0.086±0.015. The results of multivariate Cox analysis showed that liver, bone, lung, and brain metastases wereall independent prognostic factors for OS and CSS. However, according to the Finy-Gray test analysis, only bone metastasis and brain metastasis were independent prognostic factors for noncancer death. It may be that the survival time of patients with brain metastasis and bone metastasis is too short to observe the outcome of noncancer death. The height of rectal tumours affects the time to liver and lung metastasis 48. Some people believe that the higher the tumour height is, the greater the probability of liver metastasis, which may be related to the venous drainage of rectal cancer. However, we did not include the rectal cancer height variable in the analysis, so further research is necessary.

In recent years, rectal cancer has changed from a surgically managed disease into a multidisciplinary treatment model, resulting in considerable improvements in the survival and outcome of rectal carcinoma patients. A retrospective study confirmed that surgery may be overused in stage IV rectal cancer because rectal cancer surgery has decreased since 2001, but patient survival has increased (data are based on colorectal cancer cases in the past 30 years in the SEER database).

Our group of patients who choose chemoradiotherapy instead of surgery can be divided into three categories according to the purpose of treatment:

1. Patients with preoperative radiotherapy and chemotherapy had not received surgery by the end of the follow-up.

2. Patients who refuse surgery due to physical reasons or worry about postoperative complications. Due to postoperative complications of rectal cancer.

3. Intentional watch-and-wait.

The overall 1-, 3-and 5-year survival rates of our patients were 0.657, 0.266 and 0.016, respectively. According to the diagnosis time, the patients were divided into two groups: 2004-2009 and 2010-2016. The statistical description results showed that the difference in the survival rate between different diagnosis times was statistically significant (*p*< 0.001). This shows that the diagnosis time has a certain impact on the survival of patients, and it is also the development of radiotherapy and chemotherapy in recent years, which improves the prognosis of patients with rectal cancer.

There were some limitations in our study. First, this study is limited by the retrospective design and the use of large multiagency databases. This means that it may be affected by potential coding errors between different institutions and different clinical practices. Second, all the patients were from the United States, so the results may not apply to other populations. Future large-scale multi-center prospective studies are further needed to confirm the results demonstrated in the present analysis.

## Conclusion

In conclusion, we showed that age, tumour grade, T and M stage, bone, brain, liver and lung metastases, marital status and insurance status are independent risk factors for the overall survival of rectal adenocarcinoma patients who have received chemoradiotherapy but have not undergone surgery. In addition, age, tumour grade, T stage, M stage, bone, brain, liver, lung metastases and race were independent prognostic factors of CSS. Meanwhile, age, T, N and M stage, liver and lung metastases, marital status and insurance status were independently associated with noncancer-related death. We established two nomograms to facilitate visual prediction of 1- and 3-year OS and CSS based on individual clinical characteristics. Although both internal and external validation demonstrated the reliability of the nomogram, further studies are warranted. Finally, we strongly recommend rectal cancer screening for patients under the age of 50.

## Figures and Tables

**Table 1:** Clinical and demographic characteristics of the patient cohort.

Characteristic	All patients (N=3599)	Percent	Univariable				
			1-year OS (%)	3-year OS (%)	5-year OS (%)	Median survival	p value (univariate analysis)
**Age**							
18-49	570	15.8	0.697±0.020	0.287±0.022	0.178±0.024	22.000(20.116-23.884)	<0.001
50-59	914	25.4	0.715±0.015	0.294±0.017	0.184±0.018	23.000(21.447-24.553)	
60-100	2115	58.8	0.651±0.010	0.249±0.011	0.143±0.010	18.000(16.966-19.034)	
**Race**							0.036
White	2777	77.2	0.675±0.009	0.271±0.010	0.157±0.010	19.000(18.018±19.982)	
Black	455	12.6	0.681±0.022	0.211±0.021	0.125±0.021	20.000(18.308-21.692)	
Others	367	10.2	0.700±0.024	0.301±0.028	0.213±0.028	24.000(20.779-27.221)	
**Sex**							0.029
Female	1324	36.8	0.614±0.013	0.254±0.013	0.127±0.016	27.695(26.076-29.314)	
male	2275	63.2	0.700±0.024	0.301±0.028	0.213±0.028	28.979(27.769-30.189)	
**Grade**							<0.001
Grade I	236	6.6	0.752±0.028	0.313±0.034	0.201±0.036	24.000(21.091-26.909)	
Grade II	2221	61.7	0.704±0.010	0.296±0.011	0.169±0.012	22.000(20.896-23.104)	
Grade III	393	10.9	0.426±0.025	0.132±0.019	0.048±0.022	11.000(9.904-12.096)	
Grade IV	21	0.6	0.429±0.018	-	-	8.000(4.411-11.589)	
Unknown	728	20.2	0.624±0.018	0.238±0.017	0.145±0.017	19.000(17.405-20.595)	
**AJCC TNM stage**							<0.001
I	313	8.7	0.721±0.026	0.397±0.032	0.212±0.032	28.000(25.545-30.455)	
II	881	24.5	0.748±0.015	0.406±0.018	0.229±0.019	28.000(25.842-30.158)	
III	903	25.1	0.769±0.014	0.442±0.019	0.303±0.023	31.000(27.907-34.093)	
IV	1502	41.7	0.526±0.013	0.059±0.007	0.020±0.006	13.000(12.205-13.795)	
**AJCC T stage**							<0.001
T1	496	13.8	0.635±0.022	0.165±0.019	0.074±0.017	17.000(14.886-19.114)	
T2	213	5.9	0.772±0.029	0.503±0.038	0.313±0.043	38.000(26.479-49.521)	
T3	1882	52.3	0.717±0.010	0.362±0.013	0.217±0.014	25.000(23.515-26.485)	
T4	579	16.1	0.575±0.021	0.126±0.016	0.066±0.016	15.000(13.775-16.225)	
TX	429	11.9	0.522±0.024	0.036±0.011	-	13.000(11.689-14.311)	
**AJCC N stage**							<0.001
N0	1737	28.8	0.680±0.011	0.295±0.012	0.161±0.012	22.000(20.744-23.256)	
N1/2	1616	33.1	0.668±0.012	0.277±0.013	0.189±0.014	19.000(17.606-20.394)	
Nx	246	3.7	0.428±0.032	0.015±0.008	-	11.000(9.731-12.269)	
**AJCC M stage**							<0.001
M0	2097	58.3	0.753±0.010	0.419±0.012	0.258±0.013	29.000(27.269-30.731)	
M1	1502	41.7	0.526±0.013	0.059±0.007	0.020±0.006	13.000(12.205-13.795)	
**Lung metastasis**							<0.001
NO	340	9.4	0.771±0.009	0.444±0.012	0.309±0.014	30.000(27.822-32.178)	
Yes	2095	58.2	0.515±0.027	0.059±0.015	-	13.000(11.437-14.563)	
Unknown	1164	32.3	0.495±0.015	0.058±0.007	0.007±0.003	12.000(11.054-12.946)	
**Osseous metastasis**							<0.001
NO	104	2.9	0.749±0.009	0.405±0.012	0.279±0.013	27.000(25.533-28.467)	
Yes	2335	64.9	0.426±0.049	-	-	11.000(8.396-13.604)	
Unknown	1160	32.2	0.495±0.015	0.058±0.007	0.007±0.003	12.000(11.054-12.946)	
**Brain metastasis**							<0.001
NO	24	0.7	0.740±0.009	0.399±0.011	0.271±0.013	27.000(25.550-28.450)	
Yes	2413	67.0	0.167±0.076	-	-	7.000(6.225-7.775)	
Unknown	1162	32.3	0.495±0.015	0.059±0.007	0.007±0.003	12.000(11.047-12.953)	
**Liver metastasis**							<0.001
NO	582	16.2	0.780±0.010	0.483±0.013	0.342±0.016	15.000(13.816-16.184)	
Yes	1861	51.7	0.585±0.021	0.086±0.015	-	33.000(30.340-35.660)	
Unknown	1156	32.1	0.497±0.015	0.058±0.007	0.007±0.003	12.000(11.057-12.943)	
**Marital status**							<0.001
Married	1675	46.5	0.688±0.011	0.289±0.013	0.159±0.013	22.000(20.767-23.233)	
Single	1743	48.4	0.804±0.030	0.337±0.041	-	25.000(20.627-29.373)	
Unknown	181	5.0	0.612±0.012	0.237±0.011	0.140±0.012	17.000(15.983-18.017)	
**Insurance status**							<0.001
Insured	2368	65.8	0.697±0.010	0.329±0.011	0.205±0.012	23.000(21.688-24.312)	
Uninsured	1022	28.4	0.561±0.016	0.141±0.012	0.075±0.010	15.000(13.740-16.260)	
Unknown	209	5.8	0.682±0.033	0.239±0.032	-	20.000(15.704-24.296)	
**Size**							<0.001
<5mm	2168	60.2	0.683±0.010	0.302±0.011	0.192±0.012	22.000(20.845-23.155)	
≥5mm	1431	39.8	0.618±0.013	0.214±0.012	0.116±0.011	17.000(15.989-18.011)	
**Year of diagnosis**							<0.001
2004-2009	1147	32.2	0.496±0.015	0.058±0.007	0.006±0.002	12.000(11.047-12.953)	
2010-2016	2452	67.8	0.733±0.009	0.387±0.011	0.267±0.013	26.000(24.562-27.438)	

**Table 2:** The 1-year, 3-year and 5-year survival rate and median survival time in patients with different tumour stages.

Characteristic	All patients (N=3599)	1-year OS (%)	3-year OS (%)	5-year OS (%)	Median survival
**All patients**	3599	0.657+0.008	0.266+0.008	0.016+0.008	20.000 (19.179-20.821)
**Early stage (T1-2N0M0)**	313	0.721+0.026	0.397+0.032	0.200+0.032	28.000 (25.545-30.455)
**Locally advanced stage** **(T3-4N0M0; T1-4N1-2M0)**	1784	0.759+0.010	0.423+0.013	0.261+0.015	29.000 (27.032-30.968)
**Late stage (T1-4N0-2M1)**	1502	0.526+0.013	0.059+0.007	0.020+0.006	13.000 (12.205-13.795)

**Table 3:** Multiple COX regression results of OS and CSS.

OS				CSS			
Independent prognostic factors	OR	95%CI	*p*	Independent prognostic factors	OR	95%CI	*p*
**Age at diagnosis (year)**			<0.001	**Age at diagnosis (year)**			<0.001
18-49	1			18-49	1		
50-59	0.958	0.842-1.091	0.519	50-59	0.973	0.855-1.108	0.683
60-100	1.557	1.387-1.747	0.000	60-100	1.589	1.417-1.783	<0.001
**AJCC T stage**			<0.001	**AJCC T stage**			<0.001
T1	1			T1	1	0.461-0.703	
T2	0.583	0.472-0.720	<0.001	T2	0.569	0.727-0.918	<0.001
T3	0.822	0.731-0.923	0.001	T3	0.817	1.128-1.475	0.001
T4	1.273	1.113-1.457	<0.001	T4	1.290	0.881-1.178	<0.001
Tx	1.001	0.865-1.157	0.994	Tx	1.019	1.516-1.883	0.804
**AJCC M stage**			<0.001	**AJCC M stage**			<0.001
M0	1			M0	1		
M1	1.797	1.613-2.003	0.410	M1	1.690	1.516-1.883	
**Bone metastasis**			<0.001	**Bone metastasis**		1.380-2.237	<0.001
Yes	1			Yes	1		
No	0.567	0.455-0.708	<0.001	No	0.570	0.457-0.711	<0.001
Unknown	1.119	0.486-2.575	0.791	Unknown	1.071	0.475-2.416	0.869
**Lung metastasis**			<0.001	**Lung metastasis**			<0.001
Yes	1	1.321-2.058		Yes	1	0.544-0.725	
No	0.652	0.565-0.753	<0.001	No	0.628	0.402-1.586	<0.001
Unknown	0.976	0.486-1.959	0.945	Unknown	0.798		0.520
**Brain metastasis**			<0.001	**Brain metastasis**			<0.001
Yes	1			Yes	1		
No	0.297	0.195-0.453	<0.001	No	0.261	0.171-0.398	<0.001
Unknown	0.244	0.099-0.605	0.002	Unknown	0.221	0.090-0.540	0.001
**Liver metastasis**			<0.001	**Liver metastasis**			<0.001
Yes	1			Yes	1		
No	0.598	0.523-0.684	<0.001	No	0.596	0.521-0.682	<0.001
Unknown	0.752	0.325-1.740	0.506	Unknown	0.950	0.407-2.216	0.905
**Grade**			<0.001	**Grade**			<0.001
Grade I	1			Grade I	1		
Grade II	1.172	0.994-1.381	0.059	Grade II	1.152	0.977-1.357	0.092
Grade III	2.312	1.909-2.800	<0.001	Grade III	2.270	1.875-2.749	<0.001
Grade IV	3.482	2.179-5.563	<0.001	Grade IV	3.320	2.079-5.301	<0.001
Unknown	1.345	1.126-1.607	0.001	Unknown	1.316	1.102-1.572	0.002
**Marital status**			<0.001	**Race**			0.034
Married	1			White	1		
Single	0.897	0.735-1.095	0.286	Black	0.971	0.866-1.089	0.619
Unknown	1.321	1.217-1.433	<0.001	Others	0.839	0.733-0.961	0.011
**Insurance status**			0.005				
Insured	1						
Uninsured	1.148	1.049-1.257	0.003				
Unknown	1.175	0.993-1.391	0.061				

**Table 4:** Multiple COX regression results of Noncancer-related death.

Noncancer-related death			
Independent prognostic factors	OR	95%CI	*p*
**AJCC T stage**			<0.001
T1	1		
T2	0.548	0.429-0.699	<0.001
T3	0.830	0.728-0.945	0.005
T4	1.394	1.205-1.613	<0.001
Tx	1.063	0.907-1.247	0.450
**Lung metastasis**			<0.001
Yes	1		
No	0.613	0.528-0.710	<0.001
Unknown	1.078	0.593-1.961	0.805
**Liver metastasis**			<0.001
Yes	1		
No	0.602	0.522-0.693	<0.001
Unknown	1.014	0.561-1.832	0.963
**AJCC N stage**			0.007
N0	1		
N1/2	1.149	1.048-1.261	0.003
Nx	1.172	0.998-1.375	0.052
**AJCC M stage**			<0.001
M0	1		
M1	2.157	1.913-2.431	
Age			<0.001
18-49	1		
50-59	0.936	0.819-1.071	0.335
60-100	1.423	1.261-1.605	<0.001
**Marital status**			<0.001
Married	1		
Single	0.911	0.736-1.126	0.389
Unknown	1.339	1.227-1.461	<0.001
**Insurance status**			0.019
Insured	1		
Uninsured	1.129	1.023-1.246	0.016
Unknown	1.189	0.996-1.418	0.055
**Age at diagnosis (year)**			<0.001
18-49	1		
50-59	0.936	0.819-1.071-	0.335
60-100	1.423	1.261-1.605	<0.001

## Abbreviations

SEER: Surveillance, Epidemiology and End Results
OS: overall survival
CSS: cancer-specific survival
AJCC/UICC: American Joint Committee on Cancer/International Union Against Cancer
TNM: tumour-node-metastasis
C-index: concordance index
